# One year structural and functional glaucoma progression after trabeculectomy

**DOI:** 10.1038/s41598-020-59792-9

**Published:** 2020-02-18

**Authors:** Jacqueline Chua, Aistė Kadziauskienė, Damon Wong, Rimvydas Ašoklis, Eugenijus Lesinskas, Nguyen Duc Quang, Rachel Chong, Bingyao Tan, Michaël J. A. Girard, Jean Martial Mari, Jonathan G. Crowston, Tin Aung, Leopold Schmetterer

**Affiliations:** 1 Singapore Eye Research Institute, Singapore National Eye Centre, Singapore, Singapore; 2 Academic Clinical Program, Duke-NUS Medical School, Singapore, Singapore; 3 Clinic of Ears, Nose, Throat and Eye Diseases, Institute of Clinical Medicine, Faculty of Medicine, Vilnius University, Vilnius, Lithuania; 4 Department of Eye Diseases, Vilnius University Hospital Santaros Klinikos, Vilnius, Lithuania; 5 SERI-NTU Advanced Ocular Engineering (STANCE) Program, Singapore, Singapore; 6 Department of Ophthalmology, Lee Kong Chian School of Medicine, Nanyang Technological University, Singapore, Singapore; 7 Ophthalmic Engineering & Innovation Laboratory, Department of Biomedical Engineering, National University of Singapore, Singapore, Singapore; 8 GePaSud Laboratory, University of French Polynesia, Tahiti, French Polynesia; 9 Yong Loo Lin School of Medicine, National University of Singapore, Singapore, Singapore; 10 Department of Clinical Pharmacology, Medical University of Vienna, Vienna, Austria; 11 Center for Medical Physics and Biomedical Engineering, Medical University of Vienna, Vienna, Austria

**Keywords:** Diagnostic markers, Optic nerve diseases

## Abstract

We evaluated the changes in visual field mean deviation (VF MD) and retinal nerve fibre layer (RNFL) thickness in glaucoma patients undergoing trabeculectomy. One hundred patients were examined with VF and spectral-domain optical coherence tomography (OCT) before trabeculectomy and 4 follow-up visits over one year. Linear mixed models were used to investigate factors associated with VF and RNFL. VF improved during the first 3 months of follow-up (2.55 ± 1.06 dB/year) and worsened at later visits (−1.14 ± 0.29 dB/year). RNFL thickness reduced by −4.21 ± 0.25 µm/year from 1st month of follow-up. Eyes with an absence of initial VF improvement (β = 0.64; 0.30–0.98), RNFL thinning (β = 0.15; 0.08–0.23), increasing intraocular pressure (IOP; β = −0.11; −0.18 to −0.03) and severe glaucoma (β = −10.82; −13.61 to −8.02) were associated with VF deterioration. Eyes with VF deterioration (β = 0.19; 0.08–0.29), increasing IOP (β = −0.09; −0.17 to −0.01), and moderate (β = −6.33; −12.17 to −0.49) or severe glaucoma (β = −19.58; −24.63 to −14.52) were associated with RNFL thinning. Changes in RNFL structure and function occur over a 1-year follow-up period after trabeculectomy. Early VF improvement is more likely to occur in patients with mild/moderate glaucoma, whereas those with severe glaucoma show greater decline over one year. Our findings indicate that progression is observable using OCT, even in late-stage glaucoma.

## Introduction

Clinically, reduction of intraocular pressure (IOP) remains the only proven treatment for glaucoma^1–3^. Landmark glaucoma randomized clinical trials have confirmed the value of lowering IOP in delaying visual field (VF) progression^1–5^. Interestingly, recent studies^6–8^ have reported that the surgical lowering of IOP in glaucoma can result in the improvement of VF, which lends support to the preliminary data reported by Katz and Spaeth, who demonstrated reversal of structural change and field loss in newly diagnosed patients after commencement of treatment^9,10^.

Apart from functional changes, others have examined structural changes after trabeculectomy and their potential association with functional changes. The association between structural glaucomatous damage with VF decline after trabeculectomy is complex. The MoreFlow Medical Research Council 5-Fluorouracil (5-FU) study reported that out of 250 eyes, 20 eyes showed structural progression, 35 eyes showed VF progression, and 15 eyes showed both over a five years period^11^. In the Collaborative Initial Glaucoma Treatment Study (CIGTS), VF worsening was significantly associated with enlargement of the optic cup, but reversal of cupping was not associated with improvement of the VF^12^. Other studies reported an early increase in retinal nerve fiber layer (RNFL) thickness after trabeculectomy, but did not investigate the relation with VF changes^13–17^. Also, these studies have used the older time-domain optical coherence tomography (OCT)^14,16^, stereoscopic optic disc photographs^12,15^ scanning laser polarimetry^17^, or Heidelberg Retina Tomography (HRT)^11,13^, which required a manually drawn contour line and thus was more prone to operator induced variability than the newer spectral-domain OCT.

The purpose of the current study was to assess the impact of trabeculectomy and associated IOP lowering on rates of VF and RNFL thickness progression, and secondly to determine factors related to these changes following trabeculectomy.

## Results

**Figure 1:**
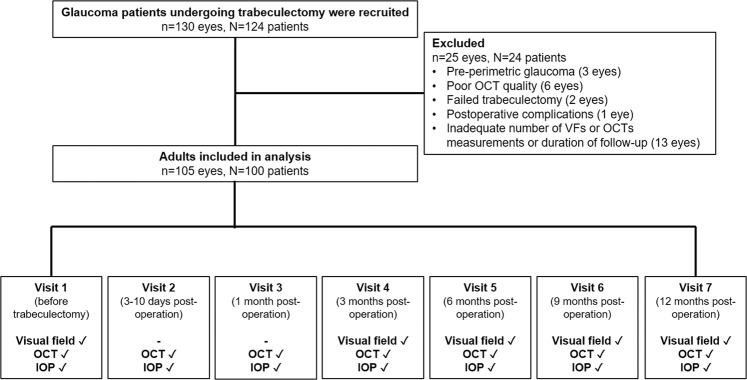
Identification of eligible glaucoma patients who underwent trabeculectomy. Of the 124 patients enrolled, 100 were included in the analysis. Of which, majority were examined at 7 visits whereas 10 missed one follow-up visit. Visual field tests were performed for 5 visits (visit 1, 4–7) and OCT/IOP were performed at all 7 visits.

Initially, 130 glaucomatous eyes of 124 patients undergoing trabeculectomy were enrolled (Fig. 1). Of these, 24 patients (25 eyes) were excluded because of pre-perimetric glaucoma (3 eyes), poor OCT quality (6 eyes), previous failed trabeculectomy (2 eyes), postoperative complications (1 eye), and inadequate number of VFs/OCTs measurements or duration of follow-up (13 eyes). Finally, we analyzed the data of 100 patients (105 eyes). Ten of these missed one follow-up visit.

The demographic and clinical baseline characteristics of the patients are presented in Table 1. The mean (±standard deviation) age of the patients’ age was 67.28 ± 8.92 years and 47% female. Baseline VF MD value was −14.68 ± 8.43 dB (range, −7.18 to −20.55 dB), and RNFL thickness was 53.38 ± 13.62 µm (range, 43 to 60 µm). Majority of these patients had exfoliation syndrome and severe glaucoma.

**Table 1:** Patient Demographic and Clinical Characteristics.

Baseline Characteristic	Glaucoma Eyes (n = 105 Eyes, 100 Participants)
Age, years	67.28 ± 8.92
Gender (female:male)	47:53
BCVA, decimal scale	0.68 ± 0.25
Refractive error, D	−0.40 ± 1.85
Central corneal thickness, µm	519.45 ± 31.22
Axial eye length, mm	23.60 ± 0.93
IOP, mmHg	27.36 ± 6.69
VF MD, dB	−14.68 ± 8.43
Global RNFL thickness, µm	53.38 ± 13.62
**Lamina cribrosa**	
Curvatures, mm^−1^	0.59 ± 0.41
GSI	−0.62 ± 0.19
Depth, µm	451.84 ± 133.35
**Glaucoma subtype**, **n (%)**	
Pseudoexfoliative	78 (74)
Primary open-angle	19 (18)
Primary angle closure	8 (8)
**Glaucoma severity**, **n (%)**	
Mild	16 (15)
Moderate	17 (16)
Severe	72 (69)

BCVA = best-corrected visual acuity; D = diopters; dB = decibels; IOP = intraocular pressure; MD = mean deviation; RNFL = retinal nerve fiber layer; VF = visual field.

Values expressed as mean ± standard deviation, unless otherwise indicated.

**Figure 2:**
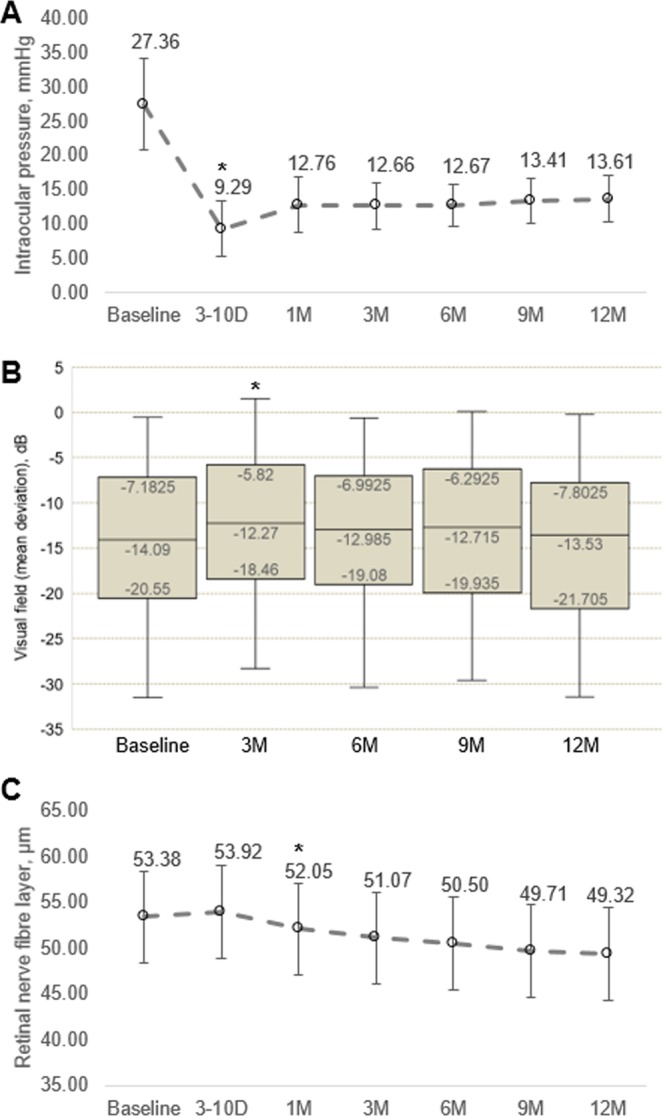
Changes in (**A**) intraocular pressure, (2) visual field mean deviation and (3) retinal nerve fibre layer thickness after trabeculectomy. *Data shown at each time point are expressed as mean and P values were obtained using comparison with the previous follow-up using linear mixed model.

The overall mean IOP before surgery was 27.36 ± 6.69 mmHg and 9.29 ± 3.98 mmHg after surgery, which represents a mean decrease of 66% (P < 0.001; Fig. 2A). VF MD improved during the first 3 months of follow-up (2.55 ± 1.06 dB/year; P = 0.016) and worsened at later follow-up visits (−1.14 ± 0.29 dB/year; P < 0.001; Fig. 2B and Table 2). RNFL thickness reduced from 1^st^ month of follow-up onwards and the mean RNFL change rate was −4.21 ± 0.25 µm/year; P < 0.001 (Fig. 2C and Table 2).

**Table 2:** Rate of visual field mean deviation and retinal nerve fibre layer thickness change after trabeculectomy.

Characteristics	Rate of Change per Year (mean ± SE)	P value
**VF MD**, **dB**		
All visits	−0.43 ± 0.22	**0**.**049**
Baseline to 3 months postoperative	2.55 ± 1.06	**0**.**016**
Postoperative 3 months to 12 months	−1.14 ± 0.29	**<0**.**001**
Global RNFL thickness, µm Baseline to 12 months	−4.21 ± 0.25	**<0**.**001**

dB = decibels; MD = mean deviation; RNFL = retinal nerve fiber layer; VF = visual field.

Linear mixed model was used to adjust for age, scan quality of OCT, follow-up duration and intracorrelation between eyes for each patient.

## Multivariate Analysis

**Figure 3:**
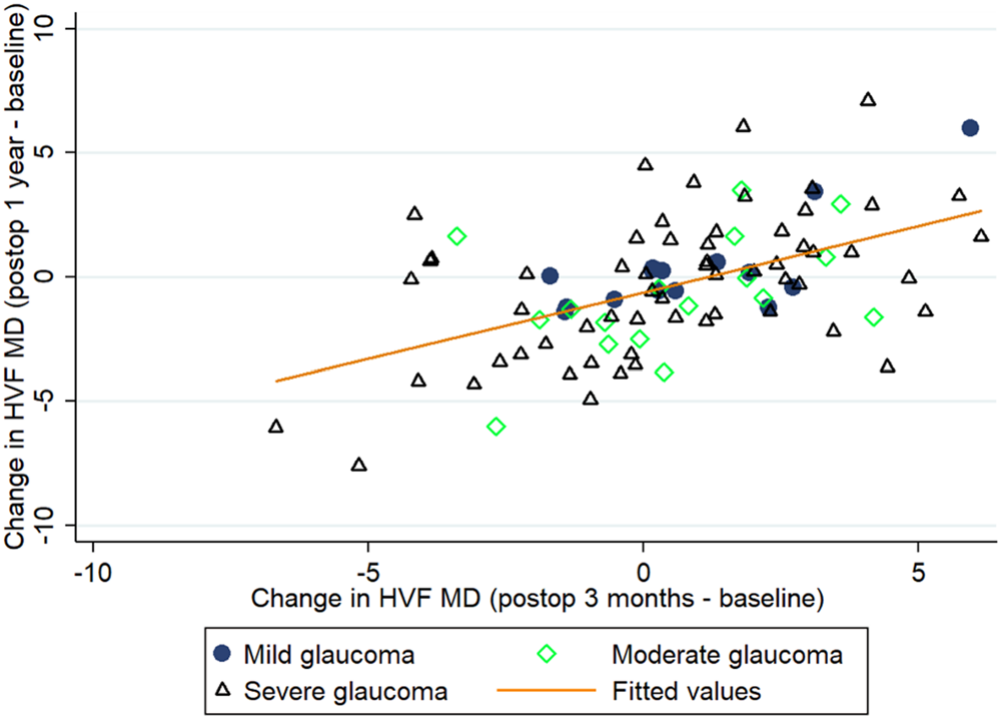
Scatterplot of the change in visual field mean deviation (VF MD) from baseline to 3^rd^ month follow-up versus the change in VF MD from 1 year after surgery compared with before, stratified by glaucoma severity. Eyes having mild glaucoma were indicated with dark filled circle, those having moderate glaucoma in green hollow diamond and severe glaucoma in black hollow triangle. The VF before trabeculectomy and the VF postoperatively (3 months after surgery) were used for the calculation of VF worsening (*r* = 0.32; P = 0.005).

Factors associated with changes in VF MD during two periods were explored: (1) baseline to 3^rd^ month (early, Tables 3) and (2) postoperative 3^rd^ month to 12^th^ month (late, Table 4). Eyes that tended to have RNFL thinning (β = 0.14, 95%CI, 0.06 to 0.23), lesser reduction in post-operatives IOP (β = −0.08, 95%CI, −0.12 to −0.04) and severe glaucoma (β = −11.88, 95%CI, −15.11 to −8.64; Table 3) were independently associated with earlier VF MD worsening. Eyes that tended to have an earlier VF MD worsening (β = 0.64, 95%CI, 0.30 to 0.98), RNFL thinning (β = 0.15, 95%CI, 0.08 to 0.23), lesser reduction in post-operatives IOP (β = −0.11, 95%CI, −0.18 to −0.03) and severe glaucoma (β = −10.82, 95%CI, −13.61 to −8.02; Table 4) were independently associated with later VF MD worsening. Figure 3 shows that those having an improvement in VF MD in early phase are less likely to have a worsening of their VF in later stages. Baseline IOP and lamina cribrosa features were not associated with VF MD change (P > 0.05; Tables 3 and 4).

**Table 3:** Factors related to changes in visual field mean deviation (Early; Baseline to 3 months postoperative).

Characteristics	Per 1 dB change in visual field mean deviation			
	Univariate		Multivariate *	
	β (95%CI)	P value	β (95%CI)	P value
Change in RNFL thickness, µm^‡^	0.29 (0.21, 0.37)	**<0**.**001**	0.14 (0.06, 0.23)	**0**.**001**
Change in IOP, mmHg^‡^	−0.05 (−0.09, −0.02)	**0**.**001**	−0.08 (−0.12, −0.04)	**<0**.**001**
Baseline IOP, mmHg	−0.17 (−0.41, 0.07)	**0**.**171**	−0.12 (−0.27, 0.02)	0.097
Central corneal thickness, µm	0.06 (0.01, 0.11)	**0**.**034**	0.03 (−0.01, 0.06)	0.105
**Glaucoma subtype**				
Pseudoexfoliative	Reference			
Primary open-angle	−2.50 (−6.73, 1.73)	0.246	—	—
Primary angle-closure	0.17 (−5.96, 6.30)	0.957	—	—
**Glaucoma severity †**				
Mild	Reference		Reference	
Moderate	−3.61 (−7.32, 0.10)	0.056	−2.60 (−5.97, 0.76)	0.129
Severe	−15.56 (−18.51, −12.61)	**<0**.**001**	−11.88 (−15.11, −8.64)	**<0**.**001**
**Lamina cribrosa**				
Changes in curvatures, mm^−1‡^	−1.08 (−2.35, 0.20)	0.099	—	—
Changes in GSI^‡^	0.93 (−3.17, 5.03)	0.658	—	—
Changes in depth, µm^‡^	−0.01 (−0.01, −0.01)	**0**.**039**	0.01 (−0.01, 0.01)	0.764

IOP = intraocular pressure; RNFL = retinal nerve fiber layer; VF = visual field.

Linear mixed model was used to adjust for patient cluster.

*Multivariate model - adjusted for age, gender, quality of OCT, change in RNFL, change in IOP, baseline IOP, central corneal thickness, glaucoma severity and change in lamina cribrosa depth in the same model.

^†^Those with severe glaucoma had −11.95 dB reduction in VF MD (95% CI, −14.83, −9.08; P < 0.001) in the univariate model and −9.27 dB reduction in VF MD (95% CI, −12.12, −6.42; P < 0.001) in the multivariate model compared with those with moderate glaucoma.

^‡^Changes in RNFL thickness (in µm) or IOP (in mmHg) or curvatures (mm −1) or GSI or depth (µm) for 1 dB improvement in visual field mean deviation at early phase.

**Table 4:** Factors related to changes in visual field mean deviation (Late; Postoperative 3 months to 12 months).

Characteristics	Per 1 dB change in visual field mean deviation			
	Univariate		Multivariate^*^	
	β (95%CI)	P value	β (95%CI)	P value
Change in VF MD, dB^‡^ (Postoperative 3 months *minus* baseline)	0.76 (0.17, 1.35)	**0**.**011**	0.64 (0.30, 0.98)	**<0**.**001**
Change in RNFL thickness, µm^‡^	0.37 (0.29, 0.44)	**<0**.**001**	0.15 (0.08, 0.23)	**<0**.**001**
Change in IOP, mmHg^‡^	−0.12 (−0.19, −0.04)	**0**.**003**	−0.11 (−0.18, −0.03)	**0**.**009**
Baseline IOP, mmHg	−0.12 (−0.37, 0.13)	0.333	−0.04 (−0.18, 0.10)	0.615
Central corneal thickness, µm	0.05 (0.01, 0.11)	**0**.**041**	0.01 (−0.03, 0.03)	0.924
**Glaucoma subtype**				
Pseudoexfoliative	Reference			
Primary open-angle	−2.49 (−6.83, 1.85)	0.261	—	—
Primary angle-closure	0.01 (−6.28, 6.30)	0.997	—	—
**Glaucoma severity**^**†**^				
Mild	Reference		Reference	
Moderate	−3.80 (−7.63, 0.04)	0.052	−2.43 (−5.37, 0.52)	0.106
Severe	−16.01 (−19.06, −12.97)	**<0**.**001**	−10.82 (−13.61, −8.02)	**<0**.**001**
**Lamina cribrosa**				
Changes in curvatures, mm^−1‡^	−0.23 (−1.72, 1.26)	0.762	—	—
Changes in GSI^‡^	0.83 (−1.22, 2.87)	0.429	—	—
Changes in depth, µm^‡^	−0.01 (−0.01, 0.01)	0.466	—	—

IOP = intraocular pressure; RNFL = retinal nerve fiber layer; VF = visual field.

Linear mixed model was used to adjust for patient cluster.

*Multivariate model - adjusted for age, gender, quality of OCT, change in RNFL, change in IOP, baseline IOP, central corneal thickness and glaucoma severity in the same model.

† Those with severe glaucoma had −12.22 dB reduction in VF MD (95% CI, −15.18, −9.25; P < 0.001) in the univariate model and −8.39 dB reduction in VF MD (95% CI, −10.82, −5.95; P < 0.001) in the multivariate model compared with those with moderate glaucoma.

^‡^Changes in early phase of VF MD (dB) or RNFL thickness (in µm) or IOP (in mmHg) or curvatures (mm^−1^) or GSI or depth (µm) for 1 dB improvement in visual field mean deviation in the late phase.

We then examined the factors associated with changes in RNFL thickness during two periods: (1) baseline to 3^rd^ month (early, Table 5) and (2) postoperative 3^rd^ month to 12^th^ month (late, Table 6). Eyes that tended to have a more pronounced worsening in VF (β = 0.26, 95%CI, 0.05 to 0.46), surprisingly, greater reduction in post-operatives IOP (β = 0.16, 95%CI, 0.11 to 0.22), pseudoexfoliative glaucoma than angle closure glaucoma (β = 13.88, 95%CI, 7.96 to 19.80), moderate (β = −9.31, 95%CI, −14.76 to −3.85) or severe glaucoma (β = −20.31, 95%CI, −25.75 to −14.87) than mild glaucoma and spherical cup shaped lamina cribrosa (β = 5.92, 95%CI, 0.53 to 11.31; Table 5) were independently associated with RNFL thinning in the early stage. Eyes that tended to have a more pronounced worsening in VF (β = 0.19, 95%CI, 0.08 to 0.29), increasing post-operatives IOP (β = −0.09, 95%CI, −0.17 to −0.01), pseudoexfoliative glaucoma than angle closure glaucoma (β = 12.65, 95%CI, 6.42 to 18.88), moderate (β = −6.33, −12.17 to −0.49) or severe glaucoma (β = −19.58, 95%CI, −24.63 to −14.52) than mild glaucoma and decreasing lamina cribrosa depth (β = 0.014, 95%CI, 0.004 to 0.025; Table 6) were independently associated with RNFL thinning in the later stage.

**Table 5:** Factors related to changes in retinal nerve fibre layer thickness (Early; Baseline to 3 months postoperative).

Characteristics	Per 1 µm change in retinal nerve fibre layer thickness			
	Univariate		Multivariate*	
	β (95%CI)	P value	β (95%CI)	P value
Change in VF MD, dB^‡^ (Postoperative baseline to 3 months)	0.74 (0.55, 0.94)	**<0**.**001**	0.26 (0.05, 0.46)	**0**.**015**
Change in IOP, mmHg^‡^	0.07 (0.04, 0.11)	**<0**.**001**	0.16 (0.11, 0.22)	**<0**.**001**
Baseline IOP, mmHg	0.09 (−0.28, 0.47)	0.621	0.18 (−0.05, 0.42)	0.125
Central corneal thickness, µm	0.06 (−0.02, 0.14)	0.122		
**Glaucoma subtype**				
Pseudoexfoliative	Reference		Reference	
Primary open-angle	−1.46 (−7.84, 4.91)	0.653	3.26 (−0.92, 7.42	0.126
Primary angle-closure	10.26 (1.00, 19.52)	**0**.**030**	13.88 (7.96, 19.80)	**<0**.**001**
**Glaucoma severity**				
Mild	Reference		Reference	
Moderate	−7.48 (−13.86, −1.10)	**0**.**022**	−9.31 (−14.76, −3.85)	**0**.**001**
Severe	−22.89 (−27.95, −17.82)	**<0**.**001**	−20.31 (−25.75, −14.87)	**<0**.**001**
**Lamina cribrosa**				
Changes in curvatures, mm^−1‡^	3.47 (2.20, 4.74)	**<0**.**001**	−2.13 (−4.49, 0.22)	0.076
Changes in GSI^‡^	5.52 (3.02, 8.01)	**<0**.**001**	5.92 (0.53, 11.31)	**0**.**031**
Changes in depth, µm^‡^	0.03 (0.02, 0.03)	**<0**.**001**	0.004 (−0.008, 0.016)	0.514

IOP = intraocular pressure; MD = mean deviation; RNFL = retinal nerve fiber layer; VF = visual field.

Linear mixed model was used to adjust for patient cluster.

^*^Multivariate model - adjusted for age, gender, quality of OCT, change in VF MD, change in IOP, baseline IOP, glaucoma severity and lamina cribrosa features (curved, GSI and depth) in the same model.

^‡^Changes in early phase of VF MD (dB) or IOP (in mmHg) or curvatures (mm^−1^) or GSI or depth (µm) for 1 µm increase in retinal nerve fibre layer thickness at early phase.

**Table 6:** Factors related to changes in retinal nerve fibre layer thickness (Late; Postoperative 3 months to 12 months).

Characteristics	Per 1 µm change in retinal nerve fibre layer thickness			
	Univariate		Multivariate^*^	
	Coefficients (95%CI)	P value	Coefficients (95%CI)	P value
Change in VF MD, dB^‡^ (Postoperative 3 months to 12 months)	0.30 (0.19, 0.40)	**<0**.**001**	0.19 (0.08, 0.29)	**0**.**001**
Change in IOP, mmHg^‡^	0.07 (0.04, 0.11)	**<0**.**001**	−0.09 (−0.17, −0.01)	**0**.**026**
Baseline IOP, mmHg	0.09 (−0.28, 0.47)	0.621	0.08 (−0.17, 0.32)	0.538
Central corneal thickness, µm	0.06 (−0.01, 0.14)	0.108		
**Glaucoma subtype**				
Pseudoexfoliative	Reference		Reference	
Primary open-angle	−1.46 (−7.84, 4.91)	0.653	2.57 (−1.96, 7.10)	0.267
Primary angle-closure	10.26 (1.00, 19.52)	**0**.**030**	12.65 (6.42, 18.88)	**<0**.**001**
**Glaucoma severity**				
Mild	Reference		Reference	
Moderate	−7.48 (−13.86, −1.10)	**0**.**022**	−6.33 (−12.17, −0.49)	**0**.**034**
Severe	−22.89 (−27.95, −17.82)	**<0**.**001**	−19.58 (−24.63, −14.52)	**<0**.**001**
**Lamina cribrosa**				
Changes in curvatures, mm^−1‡^	3.47 (2.20, 4.74)	**<0**.**001**	−0.01 (−1.57, 1.54)	0.988
Changes in GSI^‡^	5.52 (3.02, 8.01)	**<0**.**001**	1.00 (−1.17, 3.16)	0.367
Changes in depth, µm^‡^	0.03 (0.02, 0.03)	**<0**.**001**	0.014 (0.004, 0.025)	**0**.**007**

IOP = intraocular pressure; MD = mean deviation; RNFL = retinal nerve fiber layer; VF = visual field.

Linear mixed model was used to adjust for patient cluster.

^*^Multivariate model - adjusted for age, gender, quality of OCT, change in VF MD, change in IOP, baseline IOP, glaucoma severity and lamina cribrosa features (curved, GSI and depth) in the same model.

^‡^Changes in late phase of VF MD (dB) or IOP (in mmHg) or curvatures (mm^−1^) or GSI or depth (µm) for 1 µm increase in retinal nerve fibre layer thickness at late phase.

## Discussion

The current study investigated the rates of progression of VF MD and RNFL thickness and factors related to changes in the VF MD and RNFL thickness in a clinical sample of glaucoma patients undergoing trabeculectomy. After surgical reduction in IOP, we identified an initial improvement in VF followed by a subsequent decline over 9 months. We also found that the later decline in VF MD correlated with the initial changes in VF MD, degree of IOP lowering and RNFL thinning. Finally, individuals with severe glaucoma had a worse decline in both VF and RNFL thickness. Understanding VF/RNFL thickness changes over time and their related factors may be relevant when managing patients with severe glaucoma who are at greatest risk of lifetime blindness.

A critical issue is whether changes in VF over the short term as observed in the present study are real or related to fluctuations. We have demonstrated an initial VF gain after surgical intervention that could also reflect a regression to the mean (RTM) rather than an actual VF change. There are reasons to believe that the initial VF improvement is real and not simply due to variability. Firstly, our participants underwent to two VF tests at baseline which would mitigate VF measurement inaccuracy. In addition, we selected the second VF report for analysis, which should hopefully reduce learning effects and reduce measurement variability. We further ran a separate analysis using the first VF report and significance of the measured effect size and directions remained similar with the model reported in Table 3. Secondly, those individuals were measured with worse baseline VF MD did not demonstrate a more distinct improvement in MD at three-month follow-up, which is suggestive of RTM. Instead, the VF improvement was seen markedly in eyes with mild or moderate glaucoma. Finally, we perceived significant correlations between VF improvement with IOP control and RNFL thickness, which would not be likely from random fluctuation associated with RTM phenomenon The VF improvement over the 3 months after surgery was significantly related to RNFL thickness measurement, an objective and reproducible OCT biomarker, strongly indicates that the early VF improvement observed in this study is neither related to RTM nor with VF fluctuations.

Others have corroborated that VF improvement after glaucoma surgery as a real likelihood^6–9^. Spaeth was the first to report the effect of IOP lowering and the initial gain in VF following either trabeculectomy or argon laser trabeculoplasty^9^. Recent studies have reported short- and long-term VF improvement after glaucoma surgical intervention in individuals with mild to moderate glaucoma^6,7^.

We too observed an initial enhancement in VF in the first 3 months followed by a significant decline in the subsequent 9 months. This was seen even when IOP control remained good over the entire study period. However, it was important to state that higher IOP remained a risk factor for VF progression (Table 4). The most important risk factor was, however, advanced glaucoma damage with some patients losing as much as 7 dB per year. The association of this VF loss with structural progression indicates that this is not due to cataract formation after surgery. Those patients having an initial improvement in VF were less likely to experience a VF decline during the following 9 months. When comparing the VF changes over one year in the present study with those obtained in other trials^1,18–22^, the more advanced glaucomatous damage in our population needs to be considered.

An important finding is that RNFL thickness significantly declined over the one-year study period. This was seen in spite of the mostly severe glaucoma cases and the very thin RNFL thickness at baseline. Cross-sectional studies have reported the “floor effect” of RNFL thickness at approximately 50 µm with Spectralis OCT, the level at which point no additional thinning can be distinguished^23–25^. Longitudinal studies have, however, shown that structural progression can still be seen in late stage disease and that the floor effects only occur at thinner RNFL values, which is in agreement with the present study^26,27^. Also, postmortem studies suggested that a few surviving ganglion cells remain detected in cases of advanced glaucoma^28^. Hence, our data indicate that RNFL measurements can still be used to monitor VF progression in late stage glaucoma after trabeculectomy.

We^29^ and others^30,31^ have previously reported that the decline in RNFL thickness is dependent on the post-operative positioning of the lamina cribrosa. Also, we found that changes in VF were neither associated with the position or the shape of the lamina cribrosa. This makes lamina cribrosa parameters an unlikely candidate for sufficient structural progression analysis after trabeculectomy. This is in contrary to a study by Ha and co-workers, where they showed that the baseline mean anterior laminar cribrosa insertion depth was correlated with the rate of VF deterioration^32^. One plausible explanation may be related to the severity of glaucomatous damage. Ha *et al*. mostly had eyes with early glaucoma whereas ours were composed of eyes with late glaucoma. Hence, the behavior of the lamina cribrosa on VF worsening may be confounded by the glaucoma severity.

### Study strengths and limitations

Strengths of the study include the prospective study design, large number of patients with severe glaucoma and spectral-domain OCT imaging. Compared with earlier studies, spectral-domain OCT is objective and reproducible and thus less prone to operator induced variability^33,34^. Limitations of the study include the relatively short observation period, small number of patients with mild or moderate disease and the lack of cataract staging after surgery. Approximately 48% of the eyes had mild cataract, 6% were pseudophakia, whereas the remaining 46% of the eyes did not have any diagnosis of lens disorders. Even though the severity of cataract was not graded, we would like to reiterate that we only included participants with good quality OCT scans. Those with poor quality OCT scans were excluded. Moreover, most of the patients had pseudoexfoliative glaucoma and it is unclear to which degree the results can be applicable to other study populations. Last, non-standard VF reliability criteria were used (e.g. 33% false positive errors instead of 15%). We selected a wider criteria of VF reliability in order to include more patients for analysis and because many patients had late stage disease and therefore were unable to provide more reliable perimetry data. In spite of this limitation, we still observed VF changes over a period of time that were correlated to structural changes as assessed with OCT and therefore are likely to represent true changes rather than fluctuations.

In summary, we have shown that measurable changes occur in both VF MD and RNFL thickness after trabeculectomy. In the early phase, we observed an improvement in VF that was more likely to occur in patients with mild-moderate disease and less likely to occur in patients with RNFL thinning. From 3–12 months, a decline in VF was observed. Risk factors for the VF deterioration were increasing post-operatives IOP, late stage disease, RNFL thinning and absence of VF improvement in the first three months. Our data also indicate that even in late stage glaucoma, structural progression can be monitored with RNFL thickness measurement using spectral-domain OCT.

## Methods

This was a longitudinal study of consecutive patients, who were undergoing trabeculectomy at the Vilnius University Hospital Santaros Klinikos (2014–2017)^29^. Approval for conducting the study was obtained from the Vilnius Regional Biomedical Research Ethics Committee and all study procedures adhered to recommendations of the Declaration of Helsinki. Written informed consents were obtained from participants. Inclusion criteria were defined as (1) clinical diagnosis of primary or secondary glaucoma, (2) trabeculectomy indicated because of progressing glaucoma or high risk of glaucoma progression due to high IOP (defined as IOP that is higher than patient’s target IOP), (3) best corrected Snellen visual acuity of ≥ 0.1, (4) refractive error from −6.0 D to +6.0 D of sphere and ± 3.0 D of cylinder. Patients were excluded if they had any prior intraocular surgery, except phacoemulsification with intraocular lens implantation, other ophthalmological or neurological diseases affecting the VF, or poor image quality because of opaque ocular media. Glaucoma was defined based on the presence of glaucomatous optic neuropathy (neuroretinal rim thinning, notching or RNFL defects) with or without associated glaucomatous VF defect. A glaucomatous VF defect was defined as glaucoma hemifield test of standard automated perimetry outside normal limits and/or a cluster of at least three contiguous points on the pattern deviation plot with P < 5% and one with P < 1% probability of being normal or pattern standard deviation of <5%. The VF test was considered reliable if false positive and false negative errors were <33% and fixation losses <20%.

### Examination procedures

Patients underwent baseline examination including, assessment of best-corrected visual acuity and autorefractometry (Topcon KR-1 Auto Kerato-Refractometer, Topcon Medical Systems, USA), slit-lamp biomicroscopy, dilated stereoscopic examination of fundus, and partial optical coherence interferometry (IOL Master, Carl Zeiss Meditec, Dublin, CA, USA) The mean of two IOP measurements using Goldmann applanation tonometry (GAT, Haag-Streit AG, Switzerland), spaced one minute apart was calculated. If two measurements differed by more than 2 mmHg, we took a third reading, and averaged the two closest values. One ophthalmologist examined the patients.

Automated perimetry using 30–2 Swedish Interactive Threshold Algorithm Standard strategy (Humphrey visual field analyzer, Carl Zeiss Meditec, Dublin, CA, USA) were performed at five visits (before the trabeculectomy and postoperatively 3 months, 6 months, 9 months and twelve months). Baseline VF was done twice to eliminate the known learning effect and the second VF report was used for current analysis if it was reliable. Glaucoma severity was staged based on the standard automated perimetry using the Hodapp–Parrish –Anderson criteria^35^: mild glaucoma (mean deviation (MD) >−6 dB, less than 25% of points are depressed <5% and less than 10 points are depressed <1% on a pattern deviation plot, all points in the central 5° with sensitivity ≥15 dB), moderate glaucoma (MD > −12dB, less than 50% of points are depressed <5% and less than 20 points are depressed <1% on the pattern deviation plot, only one hemifield having a point in the central 5° with sensitivity <15 dB, no points within 5° of fixation with sensitivity of 0 dB), severe glaucoma (MD < −12dB, more than 50% of points are depressed < 5% and more than 20 points are depressed <1% on the pattern deviation plot, points within the central 5° with sensitivity <15 dB in both hemifields, at least one point with sensitivity of 0 dB within 5° of fixation).

All the patients underwent a limbal-based trabeculectomy, with or without adjunctive 5-fluorouracil, following the same surgical protocol by one of four surgeons. Subsequently, needling with 5-fluorouracil was performed if failure of the filtrating bleb occurred. Only patients with reduced postsurgical IOP continued the study.

### Spectral-domain optical coherence tomography

We evaluated the RNFL thickness and lamina cribrosa features using the spectral-domain OCT (Heidelberg Spectralis, Heidelberg Engineering, Dossenheim, Germany) at seven visits (before the trabeculectomy and postoperatively 3–10 days, one month, three months, six months, nine months and twelve months). A 15 × 10° rectangle scan was centered on the optic nerve head. Each OCT volume consisted of 49 serial horizontal B scans (4.5 mm long lines, 40 images averaged) spaced at approximately 63 µm intervals. At least two OCT scans were taken and the one with the best quality was chosen. Images with a quality score ≤15 were excluded. The baseline OCT scan was set as a reference and all subsequent scans were done adherent to it. Potential magnification error was avoided by entering the corneal curvature and refraction of the eye before the OCT scanning. The RNFL thickness was measured automatically from the circumferential SD-OCT scan of 3.4 mm diameter centered at the ONH (single circle B scan of 12°, 100 images averaged).

To visualize the features of the lamina cribrosa, OCT images were enhanced using adaptive compensation (Reflectivity software, version 3.4, Ophthalmic Engineering & Innovation Laboratory, National University of Singapore, Singapore) and the measurements of lamina cribrosa were extracted using Morphology 1.0 software (Ophthalmic Engineering & Innovation Laboratory, National University of Singapore, Singapore)^36–38^. Curvature was expressed as the values in mm^−1^, negative values describing posteriorly curved lamina cribrosa and positive values indicating anteriorly curved lamina cribrosa^39^. Global shape index ranged between −1 and 1 and corresponds to a transition from spherical cup (GSI = −1) through a symmetric saddle-shaped LC (GSI = 0) to spherical cap (anteriorly curved LC; GSI = 1). The mean LCD was calculated as the mean depth of all points on the surface of the lamina cribrosa.

### Statistical analyses

Baseline values were defined as those before trabeculectomy. The primary outcome variables were the postoperatively changes in VF MD and RNFL thickness. Rates of progression were calculated from longitudinal data using linear mixed models adjusting for baseline age, sex, follow-up duration, and scan quality of OCT at each visit and accounting for correlation between eyes. Associations between clinical factors (independent variables) and changes in VF MD and RNFL thickness (dependent variables) were assessed by linear mixed models to account for longitudinal data. In addition to age, sex baseline IOP, and scan quality of OCT, variables with P < 0.1 from the univariate model were included in the multivariate model. Descriptive statistics were calculated as the mean and standard deviation (SD) and number (%). We used the Wilcoxon signed-rank sum test to compare the preoperative and postoperative measurements of IOP, VF MD and RNFL thickness. A P value of less than 0.05 was considered statistically significant. Analysis was performed using STATA 12.1 (StataCorp LP, College Station, Texas).

## Data Availability

The datasets generated during and/or analyzed during the current study are not publicly available due to the terms of consent to which the participants agreed but are available from the corresponding author on reasonable request.
